# Caregivers’ Knowledge and Food Accessibility Contributes to Childhood Malnutrition: A Case Study of Dora Nginza Hospital, South Africa

**DOI:** 10.3390/ijerph182010691

**Published:** 2021-10-12

**Authors:** Pamela Clarke, Mthokozisi Kwazi Zuma, Ayuk Betrand Tambe, Liana Steenkamp, Xikombiso Gertrude Mbhenyane

**Affiliations:** 1 Department of Human Nutrition, Faculty of Medicine and Health Sciences, Stellenbosch University, Cape Town 8000, South Africa; pgnutrition@sun.ac.za (P.C.); mkzuma@sun.ac.za (M.K.Z.); ayuk.betrand@yahoo.com (A.B.T.); 2 Research Associate at the HIV & AIDS Research Unit, Nelson Mandela University, Qgeberha 6000, South Africa; Liana.Steenkamp@mandela.ac.za

**Keywords:** anthropometry, cross sectional, food security, malnutrition, children

## Abstract

Amongst the problems facing South Africa today are malnutrition and food insecurity, and there is a need for interventions and innovative strategies to address these. The aim of the study was to determine the contribution of caregivers’ knowledge of nutrition and household food security among children aged 0 to 60 months. A cross-sectional study design was applied using a quantitative approach. A convenience sample (*n* = 184) of caregiver–child pairs (for children 0 to 60 months) from the Dora Nginza Hospital Paediatric Outpatient Department was used. A structured questionnaire was applied to collect data on socio-economic factors, health status, household food security, and caregivers’ knowledge. In addition, interviews were conducted, and anthropometric measurements of children were taken to determine their nutritional status. The results indicate that most caregivers were female, and more than half completed high school, yet almost 75% were unemployed. Most of the caregivers (58.2%) were either overweight or obese. The results also show that only 33.2% of households were food secure, 29.3% were at risk of hunger, and 37.5% experienced hunger. The prevalence of stunting, underweight, and wasting among children in the study was high. A significant, slightly positive correlation was found between the body mass index of the caregiver and height for age. Poor socio-economic status and food inaccessibility were identified as possible underlying contributing factors to malnutrition, contributing to food insecurity and therefore poor dietary intake.

## 1. Introduction

Globally, one in every three people suffers from at least one form of malnutrition which may be wasting, stunting, micronutrient deficiencies, overweight or obesity, and diet-related non-communicable diseases (NCDs) [1]. Evidence also suggests that 462 million adults globally are underweight, and 1.9 billion are overweight or obese [1]. In addition, 41 million children under the age of five are overweight or obese, while 155 million are chronically undernourished [1,2,3]. According to recent studies, low- and middle-income countries (LMICs) are experiencing a rise in childhood overweight and obesity [2,3]. Furthermore, hidden hunger in the form of micronutrient deficiencies contributes to malnutrition problems in children [4]. In addition, 47% of children globally are anaemic, and 33% have vitamin A deficiency (VAD) [5].

Household hunger is decreasing in South Africa, but it is still a reality that faces many households and increases the risk of undernutrition as well as nutritional deficiencies. SANHANES-1 (2012) data showed that only 45.6% of the population were food secure, 28.3% were at risk of hunger, and 26% were food insecure (experienced hunger) [6]. The Eastern Cape was one of two provinces within which the prevalence of food insecurity was more than 30% [6]. In South Africa, food is available at the national level but presents a challenge for many households. Hunger exists in the form of hidden hunger due to poor consumption of foods, especially in low-income households [7]. Zuma et al. [8] suggest that this trend can be alleviated through improved nutrition intake or balanced nutrition derived from improved staple crops and food diversification.

There is a need for interventions and innovative strategies to improve the status of malnutrition in South Africa. Nationally, food security is improving, unlike at the individual level, where it is still lacking [9]. Various interventions have been implemented since 1998 to alleviate food insecurity, including the child support grant, pension- and disability grants, strengthened agricultural support and development, directed feeding programmes (especially within schools), and training and support via food gardens [9,10,11]. Although food consumption and dietary diversity in low-income households were found to improve after the introduction of the child support grant, the nutritional status of children has shown minimal improvement [9]. Furthermore, although social grants have shown success in reducing food insecurity, the effect on severe malnutrition is insufficient [9]. Recently, biofortification of staple crops with the aim of improving nutritional content for easily accessible foods and incorporation of indigenous underutilised foods with common diets to improve daily nutrition intake [12] were introduced.

Undernutrition in its various forms (wasting, stunting, and micronutrient deficiencies) increases children’s risk for disease and death [1,13]. Overnutrition, on the other hand, increases the risk for diet-related NCDs in later life and includes cardiovascular disease, certain cancers, and diabetes [1,13]. It is of utmost importance to decrease the prevalence of malnutrition to improve the health of children. Some contributing factors have been identified, as well as some of the interventions that have been implemented to alleviate the problem at management and national levels.

Although poverty does not always cause hunger, many research articles have shown that the primary cause of food insecurity is low income [14,15]. Poverty is often accompanied by social factors which further affect emotional wellbeing including feelings of a lack of control in life, injustice, self-worth, and stress regarding food access [16]. The inability to afford nutrient-rich food also predisposes individuals to undernutrition, as well as overweight and obesity [1,2,3].

A lack of education was also reported to further enhance the vicious cycle of poverty and malnutrition [17]. Inadequate education is considered to be one of the causes of malnutrition as also indicated on the United Nation International Emergency Fund (UNICEF) conceptual framework. A large study in Iran which investigated the causes of malnutrition showed that a higher family income and maternal education showed protective effects in stunting [18]. Children were also less likely to be stunted if the mother has secondary education [19]. A number of studies highlighted the importance of educating women and also training and equipping them to make better nutrition decisions for their families [17,20,21]. A cohort study was performed in LMICs and found that infants with low birth weight (LBW) were associated with a 2.5–3.5 times higher risk of wasting, stunting, and underweight [22]. The risk for a population with small-for-gestational-age (SGA) children to be stunted or wasted was 20% and 30%, respectively. The analysis suggested that childhood undernutrition may have its origins in the foetal period, which suggests the need to intervene as early as possible, ideally during pregnancy [22]. Interestingly, obese or overweight mothers also have an increased risk of LBW babies [23]. Maternal age can also influence malnutrition since mothers under the age of 18 years are more likely to have stunted children [19]. Teenage pregnancies and closely spaced births also increase the risk of stunting. Furthermore, pregnancies in older women are also considered higher risk pregnancies. Maternal height is also related to offspring height at all ages, and maternal height is also inversely associated with mortality, underweight, and stunting during infancy and childhood [1,24]. 

The main social determinants of malnutrition include low family income, unmarried status, and type of childcare [25]. Poor maternal care practices showed to have a relationship with malnutrition, although care practices have many components. Financial constraints can influence optimal care practices by causing insufficient food availability, insufficient funds for healthcare and sanitation and can also cause a lack of school attendance. A caregiver may also lack the knowledge regarding how to best care for a child. Furthermore, social issues or even mental health issues can hinder optimal childcare practices [25]. Mkhize and Sibanda [26] reviewed selected studies concerning the factors that affect the nutritional status of children in South Africa for 27 studies and surveys published from 2010 to 2019. Their results showed that the nutritional status of children is affected by several factors which included household food insecurity, low household income, illiterate caregivers, unemployment, inadequate dietary intake, low birth weight, (LBW) consumption of monotonous diets, poor caregiver’s nutritional knowledge, poor access to water and sanitation, poor weaning practices, age of the caregiver, and demographic characteristics of a child (age and gender).

According to South African Health Demographic Survey (SADHS) [27], 27% of children under age 5 are stunted (short for their age), 3% are wasted (thin for their height), 6% are underweight (low weight for their age), and 13% are overweight (heavy for their height). Stunting is higher among male children (30%) than among female children (25%). As shown in Table 1, Gauteng and Free State have the highest stunting prevalence (34% each), with Eastern Cape at 25%.

To identify and address specific contributing factors to malnutrition within a community, there is a need to identify contextual factors which contribute to the prevalence of malnutrition in its different forms (overnutrition, underweight, and stunting) to support intervention programmes. The aim of the study was to explore and describe the contribution of caregivers’ knowledge about nutrition and household food security in the treatment of childhood malnutrition in Dora Nginza Hospital in Eastern Cape, South Africa.

## 2. Methods

### 2.1. Study Design 

The design was a descriptive cross-sectional study with an analytical component using a quantitative approach. The prevalence of malnutrition and a description of the population will be elaborated on—which classifies the study as descriptive. An analytical study compares different exposures and the results thereof, and in this study, different contributing factors were compared for their potential effects on malnutrition, which means the study can also be described as analytical. 

### 2.2. Study Setting

The study setting was the Dora Nginza Hospital, a provincial 220-bed government hospital located in the Zwide township in the Nelson Mandela Bay (NMB) metro of the Eastern Cape, South Africa. The population size of the area is 1,195,603 with 384,794 children under the age of five years. The district health barometer indicated that 4% of childhood deaths in NMB were due to protein-energy malnutrition (PEM), 12% due to diarrhoeal disease, and 9.3% due to HIV and Tuberculosis (TB.) Severe acute malnutrition (SAM) had fatal consequences in 10.1% of cases [28]. The overweight and obesity rate of children in the NMB district was at least 6% [14]. Approximately 40 to 70 children are seen at the Paediatric Outpatient Department (POPD) department on a daily basis (1200–2100), of which approximately 6 outpatients are seen per day by the dietician (DNH statistics from the ward). 

### 2.3. Sample Size Calculation and Sampling Technique

The estimated sample size was 140 mother–child pairs using the Cochran formula [n = Z^2^ × p (1 − p)/e^2^] with a stunting prevalence of 9% in the NMB health district of the Eastern Cape [29], with a 95% confidence level and 5% precision. In addition, 14 mother–child pairs were added to compensate for the 10% non-response rate. The final sample was 184 caregiver–children (0 to 60 months) pairs. The study sample was obtained through convenient sampling. Children who met the inclusion criteria and had an accompanying caregiver who gave consent for the child and themselves to participate were recruited. All caregivers were informed about the study before data collection commenced.

### 2.4. Data Collection Procedure and Variables Measured

A convenience sample (*n* = 184) of caregiver–child pairs was used. The researcher and a trained research assistant completed a structured questionnaire which included socio-economic factors, health status, household food security, and caregiver’s knowledge. Anthropometric measurements were taken for both the child and the caregiver according to standardised methods. 

The variables measured were socio-economic and health status, household food security, caregivers’ knowledge, anthropometry (weight, height, mid-upper arm circumference (MUAC)), and growth monitoring. For the caregiver, further information was obtained regarding education level, employment, and the number of children in the caregiver’s charge. The number of people working per household, household income, as well as information regarding social grants, were also collected. The type of dwelling and the availability of water and electricity were included. 

Household food security was determined using the Household Food Insecurity Access Scale (HFIAS) [30]. This tool provided a way to determine the level of household food security in the preceding month. 

Anthropometric measurements were obtained as per standardised practice described by various authors [31,32,33]. The weight, length (for children younger than two years of age) and height (for children older than two years of age), and MUAC (for children older than six months) were obtained. Anthropometric information was obtained to provide determine the nutritional status of the participants. Z-scores describe how far and in what direction an individual’s measurements are from the reference population’s median value [33]. The WHO growth standards compare children of the same sex and age; z-scores outside of the normal range indicate a nutritional problem which could be under- or overweight. 

An electronic pan-type beam scale (Seca, model 354) was used to weigh infants younger than two years of age. This has been shown to be accurate to 0.01 kg. For children older than two years and for adults, a levelled platform electronic scale was used. This has shown accuracy to 0.1 kg. For infants, the weights were taken to the nearest gram, and for adults, to the nearest 100 g. The weight measurement was repeated twice, and the mean was recorded. If the weights differed more than 0.001 kg (infants) and 0.1 kg (older children and adults), the weights were retaken [29,30]. For infants younger than two years, a Perspex length board with a solid headboard and a movable footboard with 1 mm increments was used to measure height to the nearest 0.1 cm. These measurements were repeated twice, and the mean was recorded. In children older than two years of age and adults, a *Seca* stadiometer (model 217) was used, and the measurements were taken to the nearest 0.1 cm.

MUAC is a simple low-cost measure to assess nutritional status and is recommended by the WHO for children 6 to 59 months. A decrease in MUAC can reflect a decrease in fat- and/or muscle mass which provides a useful tool, especially when weight-for-height (WHZ) is unavailable [34]. The Road to Health Booklet (RtHB) was used to observe the growth trend of the child. 

### 2.5. Ethical Clearance

This study was granted ethical clearance by the Health Research Ethics Committee - Stellenbosch University (ethics approval: S17/10/192). Permission was also obtained from the National Department of Health, Eastern Cape Department of Health Research Committee, and the hospital. The head of the paediatric department was notified, as was the hospital sister in charge of the POPD. Primary caregivers provided informed consent for themselves and their children for participation.

### 2.6. Data Analysis

The participants were classified according to weight for age (WAZ), height for age (HAZ), and weight for height (WHZ). The weight and height of the caregivers were converted to BMI for interpretation. The anthropometric measurements were entered into the WHO anthropometry software to calculate z-scores to be used for analysis. The children were classified according to WAZ, HAZ, and WHZ. Weight for age describes body weight in relation to chronological age. WAZ < −2 is described as underweight for age. Height for age describes linear growth, and a HAZ < −2 is described as stunting. Weight for height describes the weight in relation to the height/length of the child and is used to gauge malnutrition. WHZ < −2 is described as moderate acute malnutrition (MAM), and WHZ < −3 is described as SAM. 

For HFIAS interpretation, a score of 0 to 2 indicates food security, a score of 3 to 5 indicates moderate risk or at risk of food insecurity, and a score of 6 to 9 indicates food insecurity or hunger, adjusted according to the guidelines [30].

A statistical package for the social sciences (SPSS for Windows version 25, SPSS Inc. Chicago, IL, USA) was used to analyse the quantitative data. All the data were summarised using descriptive statistics. The nutritional status of the children was categorised by using appropriate cut-offs for classification. A bivariate analysis was performed to determine the correlations between the variables. Variables were significant if the *p*-value was < 0.05. 

## 3. Results

### 3.1. Socio-Demographic Characteristics

Most caregivers were female (96.7%, *n* = 178), between 26 and 35 years (46.2 %, *n* = 85). About half of child participants were male (52.2%, *n* = 97), with ages ranging from 0 to 60 months. Most of the caregivers completed Grade 12 (52.7%, *n* = 97), and the majority were unemployed (70.1%, *n* = 129); see Table 2. Many of the caregivers had one or two children (70.6%, *n* = 130); see Table 2.

Most households had two or fewer adults (46.7%, *n* = 86) and two or fewer children (52.7%, *n* = 97) per house. The household size in NMB is reported to be an average of 3.4 people per household, showing it to be comparable to the sample. Almost a third (27.7%, *n* = 51) had nobody within the household who was working. Most children included in the study (63.6%, *n* = 117) received a monthly child support grant. A total of 137 (74%) caregivers received a grant for one or more of their children, while fewer caregivers (63.6%, n =117) received a grant for the child in the study. Other sources of household income included income from a spouse or partner (28.8%, *n* = 53), income from parents or grandparents (28.3%, *n* = 52), a child support grant (25%, *n* = 46), income from family and friends (9.2%, *n* = 17), and income from other grants (8.7%, *n* = 16). Most participants (55.5%, *n* = 102) had a household income of over 2000 ZAR per month.

### 3.2. Caregivers’ Nutrition Knowledge 

The results showed that most (87.8%, *n* = 159) of the caregivers correctly identified breastmilk as the best food for a baby younger than six months, and 32% (*n* = 58) also correctly thought that the ideal duration of breastfeeding is 24 months and beyond (Table 3). Only 24.3% (*n* = 44) correctly identified rice or bread as a suitable alternative to porridge, 9.2% (*n* = 17) identified legumes as a suitable meat alternative, and 27.7% (*n* = 50) correctly identified cheese as a suitable alternative to milk. Peanut butter was selected as a suitable milk alternative by 51.9% (*n* = 94). Most caregivers (68.5%, *n* = 124) correctly indicated that a child aged 2 to 5 years needs at least one and a half cups of milk per day. A total of 91.7% (*n* = 166) of the caregivers knew that the child would need to eat at least three times per day. For the three questions that covered breastfeeding as the best food, duration, and how often to feed their child, 70.5% (*n* = 128) of caregivers answered correctly. The clinic or community health centre was the main source of nutritional information (47.5%, *n* = 86). Other sources where nutrition knowledge was obtained from were friends and family (28.7%, *n* = 52), the media (television, radio, newspaper) (23.2%, *n* = 42), own experience (16%, *n* = 29) or the internet (5%, *n* = 9). 

### 3.3. Household Food Insecurity Measured Using Food Availability and Accessibility

Household food security was measured using HFIAS which estimates food accessibility and availability in the past month. More than half (61.4%, *n* = 113) of the caregivers or household residents had to eat a smaller variety of food due to lacking money to buy food (Table 4). Some caregivers (15.8%, *n* = 29) reported that they or another household resident had no food to eat at times, and 12.5% (*n* = 23) of the caregivers or household residents reported having had at least one member in the house sleep hungry at night due to lack of food. A few caregivers or household residents (10.3%, *n* = 19) went a whole day and night without food due to lack of resources.

**Figure 1:**
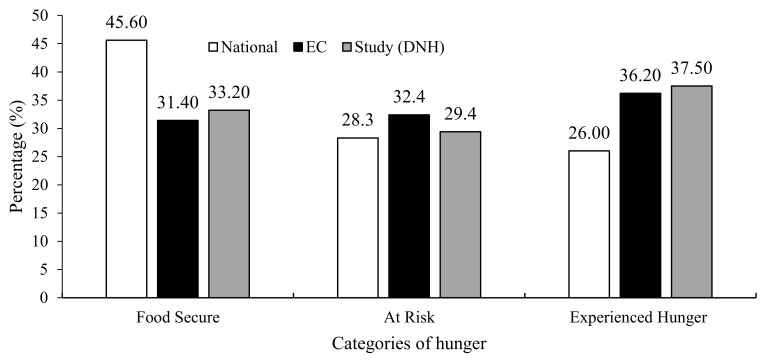
Comparison of household hunger distribution for this study and SANHANES-1.

Household hunger was also determined. As displayed in Figure 1, 33.2% of households included in the study were classified as food secure, 29.3% were at risk of hunger, and 37.5% experienced hunger. The study findings showed that most households experienced hunger (37.50%), compared with the EC (36.20%), and National (26%). 

### 3.4. Anthropometry

An interpretation of the results indicated that 2.8% (*n* = 5) of the children were classified with SAM, 3.9% (*n* = 7) with MAM, and 13.3% (*n* = 24) of the children were at risk of wasting (see Table 5). A few children (7.2%, *n* = 13) were overweight or obese. According to the WAZ, 13.6%, (*n* = 25) of the children were underweight for age. A quarter of the children (25.6%, *n* = 47) were stunted, and 12.5% (*n* = 23) were severely stunted. Even though 43 children were premature, adjusted age was not used in this study since most of the caregivers did not know the gestational age of their child. 

**Figure 2:**
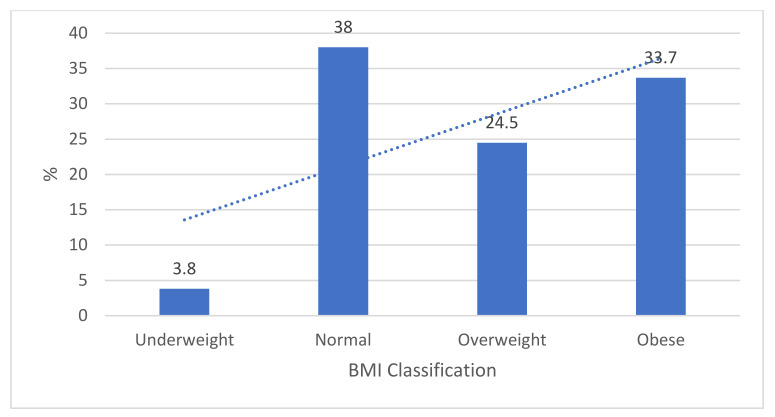
BMI classifications of caregivers.

Findings on the BMI classification of caregivers revealed that most caregivers (58.2%, *n* = 107) were either overweight or obese (Figure 2). Less than two-fifths had a normal BMI (38%, *n* = 70), and a few (3.8%, *n* = 7) participants were classified as underweight. The trend line shows that weight is on the rise to overnutrition.

### 3.5. Relationship between Socio-Demographic Characteristics, Food Security, Caregivers’ Nutrition Knowledge, and Anthropometry

Variables which have shown a significance with a *p*-value of < 0.05 are reported in Table 6. The selected variables include the anthropometry of the children and caregivers and socio-economic factors. All the investigated correlations were only slightly positive, meaning that a causal relationship could not be determined, but that value X and value Y would agree in terms of being low, moderate, or high. In general, larger caregivers were often seen with bigger children, a higher household income was seen with an improved nutritional status in children, and MUAC increased or decreased according to the size of the child.

Height for age showed a weak positive correlation with MUAC (*p* = 0.0001) and BMI of caregivers (*p* = 0.009) (Table 6). This would indicate that taller children were likely to have a larger MUAC, or children with a larger MUAC were generally taller. This could also indicate the opposite—namely, that children who are shorter generally have a smaller MUAC. Caregivers with a higher BMI tend to have children who are taller and caregivers with a lower BMI may, in general, have shorter children. 

The WHZ showed a weak positive correlation with MUAC (p = 0.000) and the BMI of caregivers (*p* = 0.0001). Therefore, caregivers with a higher BMI tend to have larger children (with a higher WHZ), and caregivers with a lower BMI tend to have thinner children (children with a lower WHZ). WHZ also showed a correlation between MUAC (0.000), meaning an increase in WHZ is associated with an increase in MUAC. This is to be expected since MUAC is another indicator for acute malnutrition besides WHZ, and it can also be used to identify obesity. WAZ also showed a weak positive correlation between MUAC (*p* = 0.0001) and gestational age (0.038). An increase in WAZ is usually associated with a larger MUAC, and a lower WAZ is associated with a smaller MUAC. An increase in gestational age is associated with an increase in WAZ, and a lower gestational age is associated with a lower WAZ. This would be expected since a lower gestational age would often result in lower birth weight and therefore a smaller child. 

MUAC showed a weak positive and significant correlation with the child’s appetite (*p* = 0.000), whether or not the caregiver receives a grant (*p* = 0.000), and household income (*p* = 0.000). An increase in appetite would mostly be seen in children with a larger MUAC, and a decrease in appetite would often be seen in children with a smaller MUAC. The presence of a social grant would increase the household income. A higher household income showed a larger MUAC in children, and a lower household income showed a lower MUAC in children. 

**Figure 3:**
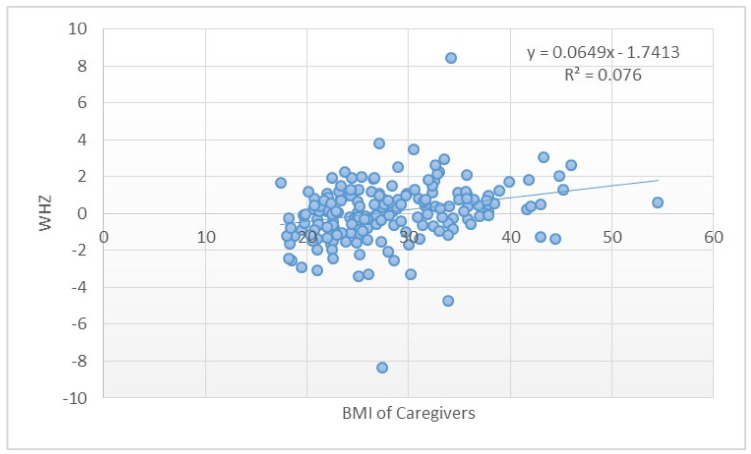
Scatter plot of the WHZ and BMI of caregivers.

**Figure 4:**
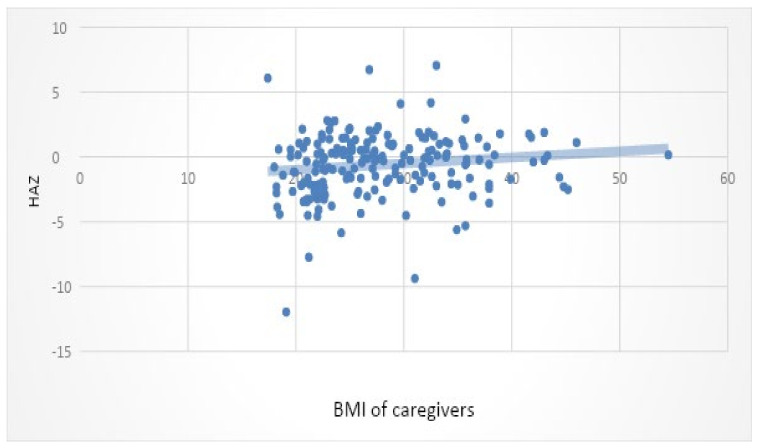
Scatter plot of HAZ and BMI of caregivers.

The WHZ of the child showed a weak positive correlation to the BMI of the caregiver (Figure 3). This could indicate that a heavier caregiver would often have a heavier child. A weak positive linear correlation was seen between HAZ and BMI of the caregivers (Figure 4). Instead of the double burden of malnutrition, which is often present within one household, heavier caregivers were seen to have heavier and taller children, while caregivers who were smaller showed to have shorter and smaller children. A weak, mild, and significant correlation between WHZ and MUAC of the child was observed. This is to be expected since MUAC can also be used as an indicator of malnutrition. An increase in WHZ should be noted with an increase in MUAC, and a decrease in WHZ should be seen with a lower WHZ. MUAC was significantly associated with HAZ and WHZ. 

## 4. Discussion

Over two-thirds of the primary caregivers were unemployed, and more than half reported that their partner or spouse were contributing sources of household income, showing that men may more frequently be the breadwinner within the households. The current findings revealed that more than half of the caregivers had one to two children per household, indicating that most households were of moderate size. Less than 10% had more than five children per household. Previous studies have reported that large households are a risk factor for malnutrition [13,35]. In this study, less than 10% would be considered to be at risk of malnutrition due to large household size.

The primary caregivers were fairly well educated, with just over half of the caregivers who completed Grade 12, 13% who had a higher education, and just over 5% did not complete primary school. Other studies have shown that the level of education of the caregiver was considered a risk factor for malnutrition, increasing the risk of underweight and stunting when parents are illiterate [17,36,37,38,39]. The current study found that less than a third of caregivers were employed, showing that a lack of higher education could hinder employment, further risking household food security and possibly increase the risk of undernutrition in children.

Household income showed to be low, with almost half that had a household income of less than 2000 (ZAR), which may not be enough to ensure livelihood since the national poverty line was set at 547 (ZAR) in 2018, which is the minimum amount needed to buy just enough food per person to meet nutritional requirements [40]. Our finding is confirmed by Tette [25], who suggested that financial constraints can influence optimal care practices by causing, amongst others, insufficient food availability. Thus, the households in this study may have had poor financial resources. Most households have four or more people which shows that more than half of the participants would not have enough money to buy sufficient food to meet nutritional requirements in a month. Almost three-quarters of the caregivers were unemployed, and almost a third had no one within the household who was working. Most caregivers were dependant on a monthly government social grant. Many studies have shown that low household income is considered a contributing factor to malnutrition [10,17,39]. When insufficient resources are available within a household, accessibility to a diverse, sufficient, and suitable diet becomes limited. When food insecurity is present, the coping mechanisms of families differ but are mostly present in terms of financial and food compromise strategies [41]. 

In the current study, less than half of households had a total income which might not be enough to ensure livelihood. A correlation was found between MUAC of the child and household income, showing that children were often more undernourished when household income was lower. It also showed a correlation between an improved nutritional status (measurable with MUAC) and a higher household income. Similarly, many studies have shown that low household income is considered a contributing factor to malnutrition [10,38].

Insufficient water sanitation and hygiene (WASH) in children is known to increase the risk of undernutrition due to an increased risk of diarrhoeal disease, intestinal parasite infections, and environmental enteropathy [19]. Previous study findings within South Africa reported that insufficient access to clean and safe water and insufficient sanitation are contributing factors to malnutrition [25,38,42]. Within a household, poor sanitation and diseases can rapidly spread among residents, leading to a vicious cycle of disease and undernutrition [25]. This statement was further strengthened by the UNICEF conceptual framework which describes insufficient access to clean water and sufficient sanitation as an underlying contributing factor to malnutrition [38]. In this study, no direct correlation was found between a lack of WASH and undernutrition. The majority of the study population (82%) had a tap with running water inside their home, and 75% had a flush toilet inside their home. This indicated that few participants were exposed to poor sanitation. Even though no correlation was found in this study, the relationship should be explored further in large study populations, as the literature has described a relationship between a lack of WASH and undernutrition. 

The study results found that caregivers received a social grant for almost two-thirds of participating children, and more than two-thirds reported receiving the grant for at least one child. A relationship was also found between receiving a social grant and MUAC of the child, showing that a presence of the social grant leads to lower undernutrition (MUAC). Social grants were first introduced 21 years ago and have been shown to alleviate some poverty [13]. Improvement in food consumption, dietary diversity, and food insecurity was noted since 1998, with an improvement in acute malnutrition but a decline in chronic malnutrition. The improvements were believed to be partly attributed to child support grants, yet the amount is still considered insufficient to cover even the basic food needs of members within a family.

Household income and the presence of receiving a social grant have shown a slightly positive correlation to the MUAC of the child. This indicates that socio-economic factors (household income) contribute to the nutritional status of the child (measured by MUAC). Household food security is present when food is available, accessible, and consumable, as well as when having a stable food supply in the household [38]. Household hunger is present when there is insufficient food available for the number of people within the house. Household hunger increases the risk of malnutrition due to the consumption of insufficient calories and nutrients and also increases the risk of eating unhealthy, non-age-appropriate foods, or foods that are not suitable for consumption any longer. This may further lead to disease which can increase the risk of malnutrition [6]. National statistics of 2011 reported that almost half a million households in the Eastern Cape have run out of money for food within the past year and almost a fifth of households reported skipping at least one meal [43]. 

Caregivers had good knowledge of the role of breastmilk for the young child and knew the duration and frequency of feeding. They also identified legumes, peanut butter, and milk as good food for 2- to 5-year-olds including the number of meals per day. Their main source of nutrition information was primary health care clinics. According to UNICEF, the causes of undernutrition and overnutrition include poverty, lack of knowledge and access to nutritious and adequate diets, poor infant and young child feeding practices, and the marketing and sales of foods and drinks can also lead to undernutrition, as well as overweight and obesity [40]. Women, in particular, mothers, have an important role to play in the eradication of malnutrition [40]. Gender inequality, manifesting in a variety of ways including smaller wages, more work, lack of maternity leave, lack of access to healthcare, and even eating after the men in some cultures, has been present for decades and is still present today. A caregiver may also lack knowledge regarding how best to care for a child. In addition, social issues and even mental health issues can hinder optimal childcare practices [25]. The caregivers in this study were knowledgeable; however, we did not measure whether this translated into good care practices.

The HFIAS questionnaire was used to identify household hunger in the present study and showed that only 33.2% of households included in the study were classified as food secure, 29.3% were at risk of hunger, and 37.5% experienced hunger. The current food security rate of 33,2% was similar to the results of the Eastern Cape (31.4%) but lower when compared with the national rate (45.6%) [6]. Household hunger was also far lower on a national level (26%), while the results in the Eastern Cape (36.2%) [6] and in this study (37.5%) were similar. The community childhood hunger identification project (CCHIP) index was used to identify childhood hunger for a national study, whereas this study used HFIAS. The results showed that 45.6% of the population were food secure, 28.3% were at risk of hunger, and 26% experienced hunger. Most hunger was experienced in rural formal and urban informal locations [6]. The participants in this study came from Ibhayi township and would have been residing in urban formal or informal settings. The type of human settlement determines the resources including water and sanitation, which can contribute to malnutrition.

According to Labadarios et al. [44], food security improved from 25% to 48% over 10 years (1999–2008), and the current study findings of 2018 showed that 33.2% were food secure. Hunger also decreased from 52.3% to 25.9% [44], while the current research study reported 37.5% to be food insecure, which is a higher score. The risk of hunger increased from 23% to 25% [44] which is slightly lower than the current study results at 29.3%. Other significant results from the HFIAS showed that almost two-thirds of caregivers worried about not having enough food, and almost half ate a small variety of food and were unable to eat more of the food they wanted. Over two-fifths reported having to eat food they did not want to eat, eating a smaller meal than what they felt they needed, or eating fewer meals a day due to lack of food. 

Interventions to improve malnutrition would therefore have to start with one of the root causes—namely, a lack of resources. Intervention programmes would, therefore, need to be mindful of a lack of resources, as well as finding ways to address it to improve accessibility to food and, consequently, food security. Overweight was also prevalent with 7.2% of children who were either overweight or obese. This was lower than the national average of 2016 at 13% and even lower than the 2013 results at 22.9% [6,27]. The results obtained could once again be different from the general public since the participants were from a hospital and already had an underlying disease condition which puts them at higher risk of undernutrition. Rural areas were also found to have an increased risk of undernutrition and the lowest obesity in children. 

The BMI of the caregivers revealed that one in four was overweight and one in three were obese, which is in line with national data reporting that more than a third of South African women are obese [6]. A slightly significant positive correlation was found between the BMI of caregivers and the WHZ and HAZ of the child. This shows that larger caregivers usually have larger children and smaller caregivers may have smaller and/or shorter children. The relationship noted could indicate the possible influence of genetics on the size of a child but could also be an indicator of socio-economic factors, since a household may not be food secure, increasing the risk of undernutrition in the caregiver and child. A correlation was found between household food security and dietary diversity, indicating that the dietary diversity would be poorer in food-insecure households, which, in turn, increases the risk of undernutrition.

The WHO also reported that children have an increased risk of obesity when they have an obese caregiver, yet simultaneous adult obesity and childhood undernutrition are common in the same community and household [1]. In this study, over- and undernutrition were noted in caregivers and children. It was seen, however, that instead of the double burden of malnutrition noted within households, the caregiver and the child had a similar nutritional status (as in overnutrition or undernutrition). A study by Dubois [45], which included data from four countries and over 24,000 children and included children from birth to 19 years, showed that the BMI of mothers was significantly and highly correlated with HAZ and BMIZ of children, showing a strong genetic component.

Genetics play an important role in the variation of weight, height, and BMI of children, although environmental factors also play a significant role [45]. These findings emphasise the importance of interventions in the family and social environment and the need to identify children who may be predisposed to obesity due to genetics. Childhood obesity is characterised by susceptibility to obesity, together with poor dietary intake and a sedentary lifestyle. Together with the risk of obesity for a child with a larger caregiver, a child may also be predisposed to undernutrition due to malnourished caregivers. The mother’s prenatal nutritional status, infections, and intestinal inflammation were also thought to contribute to malnutrition risk, showing genetics to influence many biological pathways which increase the risk of malnutrition [46]. By understanding the risk of malnutrition due to genetics, early intervention can start to reduce the risk.

Several studies have reported on HFIAS and the nutritional status of children with mixed results. HAZ was positively associated with HFIAS in some studies [47], although other studies did not find a significant relationship [48]. One of the studies conducted in Ethiopia showed that HFIAS was not associated with stunting and suggested that when households face severe food insecurity, the available resources are often shifted to the children, or stunting is a long-term consequence which does not show immediate effects. An increase in household income was shown to improve dietary intake and variety in diet and can, therefore, improve nutritional status [39]. When households are not food secure, less varied diets would be consumed.

Some of the contributing factors to malnutrition include poor socio-economic factors; specifically, a lack of further education in the caregivers, poor household income, and household food insecurity. Poor dietary practices include poor continuation of breastfeeding, a high intake of grains, and high-sugar snack foods, with a poor intake of protein-rich foods, dairy, fruit, and vegetables. It is clear that one of the driving forces of malnutrition is poor socio-economic status. This is displayed as poor household food security, leading to poor dietary diversity of children which is also significantly correlated to the nutritional status of the child. Poor food security within a household will influence the weight of the caregiver and the child, showing a caregiver and a child with a lower weight. Simultaneously, a lack of nutrition knowledge could also lead to poor food choices and excessive consumption when it is available, contributing to overweight in both the caregiver and child. 

## 5. Limitation of the Study

The sample size of the current study was relatively small. However, other studies on malnutrition in children using a hospital as a sampling frame have reported 956 sample children under five years admitted with SAM from 2014 to 2018 in public hospitals of Limpopo Province [49]. The sample represents the hospital children population. Thus, our findings are only generalisable to similar public hospitals in South Africa. Another longitudinal study conducted between March 2012 and March 2015, a birth cohort study in a periurban area called Paarl, located 60 km outside of Cape Town, South Africa also had 1004 mother–child pairs [50]. Thus, our sample size for a sample recruited in 4 weeks is comparable. Furthermore, the data were collected at an outpatient department of a hospital, which means that disease conditions may have influenced the nutritional status of the participants, and therefore, the data collected may not be completely representative of the general healthy population. The research study assumed that the caregivers answered questions truthfully and that the researchers aimed not to ask the participant leading questions which could alter their responses. 

## 6. Conclusions

The study found a number of possible contributing factors to malnutrition with children and caregivers from this area displaying similar outcomes to other underprivileged communities. More emphasis should be placed on ensuring that dietary diversity improves, but it will be difficult to comply in a food-insecure situation. The study showed the existence of stunting among children to be higher than underweight and wasting. Therefore, there is a need for nutrition education in the study location and surrounding areas. Poverty was identified as a possible underlying contributing factor to malnutrition and food insecurity and, therefore, poor dietary intake. 

## Figures and Tables

**Table 1:** Malnutrition in children 0–59 months in South Africa [27].

Province	% Stunting
Gauteng	34
Free state	34
KwaZulu-Natal	29
Northwest	27
Eastern Cape	25
Western Cape	23
Limpopo	22
Mpumalanga	22
Northern Cape	21

**Table 2:** Caregivers’ characteristics.

Characteristics of Caregivers	Caregivers (%)	Children (%)
**Participants Gender**		
Male	6 (3.3)	97 (52.7)
Female	178 (96.7)	87 (47.3)
**Ethnicity**		
African	107 (58.2)	108 (58.7)
Coloured	69 (37.5)	68 (37)
Caucasian	6 (3.3)	6 (3.3)
Indian	1 (0.5)	1 (0.5)
Other (prefer not to be classified)	1 (0.5)	1 (0.5)
**Level of School Completed**		
Grade 3 or less	3 (1.6)	-
Grade 6	9 (4.9)	-
Grade 9	51 (27.8)	-
Grade 12	97 (52.7)	-
Any tertiary education	24 (13)	-
**Age Categories (years)**		
16–25	52 (28.3)	
26–35	85 (46.2)	
36–40	18 (9.8)	
>40	29 (15.7)	
**Employment**		
Unemployed	129 (70.1)	-
Employed (part- or full-time)	48 (26.1)	-
Studying	7 (3.8)	-
**Number of Children in the Household**		
2	130 (70.6)	-
4	51 (27.8)	-
5–6	3 (1.6)	-

**Table 3:** Knowledge of caregivers regarding nutrition (*n* = 181).

Nutrition Knowledge of Caregiver	Frequency (n)	Percentage (%)
**Which food is the best for a baby younger than 6 months?**		
Breastmilk (correct answer)	159	87.8
Infant formula	3	1.7
Breast- and formula milk	12	6.6
Soft porridge	6	3.3
Do not know	1	0.6
**Ideal duration of breastfeeding**		
24 months + (correct answer)	58	32
< 6 months	21	11.7
6 to 12 months	34	18.8
12 to 18 months	33	18.2
19 to 23 months	31	17.1
Do not know	4	2.2
**Suitable substitute for porridge**		
Rice or bread (correct answer)	44	24.3
Meat or milk	17	9.4
Banana or mango	30	16.6
Cabbage or pumpkin	85	47
Do not know	5	2.7
**Suitable meat alternative**		
Legumes (correct answer)	17	9.2
Spinach	60	32.6
Potatoes	101	54.9
Do not know	3	1.6
**Amount of dairy needed/day for children 2 to 5 years**		
1.5 to > 2.5 cups (correct answer)	124	68.5
0.5 to 1 cup	29	16
Do not know	28	15.5
**Suitable milk alternative**		
Cheese (correct answer)	50	27.7
Coffee creamer	18	9.9
Peanut butter	94	51.9
Do not know	19	10.5
**Number of times a child of 2 to 5 years old should eat per day**		
Three or more (correct answer)	166	91.7
Once	1	0.6
Twice	10	5.5
Do not know	4	2.2
**Sources of nutrition information (more than one option was allowed**		
Clinic/community health centre	86	47.5
Friends/family	52	28.7
Television, radio, newspaper, magazine	42	23.2
Own experience	29	16
Internet	9	5

**Table 4:** Household food insecurity scale results breakdown per question (*n* = 184).

Household Food Insecurity Scale Questions	Frequency (n) (‘yes’ answers)	Percentage (%)
During the past 30 days:		
Did you worry that your household would not have enough food?	113	61.4
Were you or a household member unable to eat the types of food you like more?	86	46.7
Did you or a household member have to eat a small variety of food?	90	48.9
Did you or a household member have to eat foods that you really did not want to eat?	76	41.3
Did you or a household member have to eat a smaller meal than you needed?	77	41.8
Did you or a household member have to eat fewer meals in a day?	67	36.4
Was there ever no food to eat due to lack of resources?	29	15.8
Did you or a household member sleep hungry because of lack of food?	23	12.5
Did you or a household member go a whole day and night without eating anything because there was no food?	19	10.3

**Table 5:** Anthropometric classification of surveyed children (*n* = 184).

Anthropometry as per Z-Scores	Frequency (*n*)	Percentage (%)
**Weight for age (WAZ)**		
Severely underweight for age, < −3	13	7.1
Moderate underweight for age, −3 to < −2	12	6.5
Normal, ≥ −2	159	86.4
**Height for age (HAZ)**		
Severely stunted, < −3	23	12.5
Moderately stunted, −3 to < −2	24	13.1
Normal, ≥ −2	137	74.4
Weight for height (WHZ) * (*n* = 181)		
SAM, < −3	5	2.8
MAM, −3 to < −2	7	3.9
At risk of wasting, −2 to ˂ −1	24	13.3
Normal, −1 to ˂ 1	104	57.4
At risk of overweight, 1 to ˂ 2	28	15.4
Overweight, 2 to < 3	10	5.5
Obese, > 3	3	1.7
**MUAC (*n* = 106)**		
MAM, (11 to 12.5 cm)	4	4
At risk of malnutrition, (12.5 to 13.5 cm)	11	10
Normal, (> 13.5 cm)	91	86

* three values missing due to child too small to determine WHZ.

**Table 6:** Correlations between socio-demographic characteristics, dietary patterns, food insecurity, and anthropometry.

Variables		Pearson-r	*p*-Value
HAZ	MUAC	0.14	0.0001 ***
	BMI of caregivers	0.018	0.009 **
WHZ	MUAC	0.23	0.000 ***
	BMI of caregivers	0.08	0.0001 ***
WAZ	MUAC	0.30	0.0001 ***
	Gestational age	0.02	0.038 *
MUAC	Child’s appetite	0.01	0.000 ***
	Receiving grant	0.01	0.000 ***
	Household income	0.004	0.000 ***

Level of significance * *p* = 0.01, ** *p* = 0.001, *** *p* = 0.0001.

## Data Availability

The data presented in this study are available on request from the corresponding author. The data are not publicly available due to using databases that are not online.
